# TPPP3 Associated with Prognosis and Immune Infiltrates in Head and Neck Squamous Carcinoma

**DOI:** 10.1155/2020/3962146

**Published:** 2020-10-06

**Authors:** Zheng Yang, Xiaohong Li, Jingyu Li, Qisheng Su, Yuling Qiu, Zunni Zhang, Liqian Zhang, Wuning Mo

**Affiliations:** ^1^Department of Clinical Laboratory, First Affiliated Hospital Guangxi Medical University, Nanning, Guangxi Zhuang Autonomous Region, China; ^2^Department of Otorhinolaryngology and Head and Neck Surgery, First Affiliated Hospital Guangxi Medical University, Nanning, Guangxi Zhuang Autonomous Region, China

## Abstract

Tubulin polymerization promoting protein family member 3 (TPPP3) is a kind of protein that can mediate the dynamics and stability of microtubules. However, the correlations of TPPP3 between prognosis and immune infiltrates in different tumors are still unclear. The analysis of TPPP3 expression was performed via Oncomine and Gene Expression Profiling Interactive Analysis (GEPIA) website. We also used GEPIA to assess the impact of TPPPT3 on clinical outcomes. The related pathways involved in TPPP3 were analyzed by gene-set enrichment analysis (GSEA), and the correlation between TPPP3 and immune infiltration was studied by Tumor Immune Estimation Resource2.0 (TIMER 2.0). The TPPP3 expression was significantly reduced in head and neck squamous carcinoma (HNSC) compared to adjacent tissues. In addition, the low expression of TPPP3 in HNSC was significantly associated with prognosis. The pathways closely related to the low expression of TPPP3 are “Antigen Processing and Presentation,” “Primary Immunodeficiency,” and so on. The TPPP3 expression was negatively correlated with the level of CD8+ T cell, B cell, and myeloid dendritic cell infiltration in HNSC. The TPPP3 expression is closely related to multiple immunomarkers in CD8+ T cell and Myeloid dendritic cells. These data indicate that TPPP3 is associated with multiple cancers and involves multiple immune-related pathways, and TPPP3 is associated with immune infiltration levels. Besides, the TPPP3 expression may help regulate tumor-associated CD8 + T cells, DC cells in HNSC. We conclude TPPP3 can be considered as a biomarker for predicting head and neck squamous cell carcinoma prognosis and immune infiltration.

## 1. Introduction

Head and neck squamous carcinoma (HNSC) is one of the most frequent tumors in Southeast Asia and southern China. The mechanism of HNSC development is complex and involves the alteration of polygenic and multisignaling pathways [1]. During this process, viral factors, environmental factors, and genetic factors affect tumor-related gene regulation and abnormal expression [2–5]. The early diagnosis of HNSC is difficult due to the hidden physiological position of HNSC, and it is prone to lymph node and distant metastasis. Therefore, screening biomarkers are helpful for the diagnosis and pathological indicators of tumorigenesis and development.

TPPP3, also known as TPPP/p20, is a protein-coding gene located on chromosome 16 that was first reported in 2006. TPPP3 is a brain-specific protein homologous to TPPP/p25, expressed in many human cells and organs, which could induce tubulin polymerization and microtubule (MT) bundling [6]. Microtubules are the main components of mitotic spindles, which control all aspects of cell division and chromosome separation. Studies have shown that microtubule dynamics change in cancer cell division and are associated with the development of chromosomal instability, anaplasia, and drug resistance [7]. Many kinds of tumor drugs exert their anticancer effect by acting on microtubules and microtubule-related proteins. The classical anticancer drugs such as vincristine and paclitaxel are widely used in the clinic, so the further exploration of microtubules and tubulin is of great significance to the study of tumor prevention and treatment.

A growing number of researchers in recent years have begun to explore the relationship between TPPP3 and tumors. Studies suggest that reduced TPPP3 can lead to abnormal mitoses, such as the formation of multipolar spindles and chromosome segregation errors, leading to HeLa apoptosis [8]. However, the potential role of TPPP3 in HNSC development or metastasis remains unknown.

In the tumor microenvironment, immune and stromal cells are two major types of nontumor components. The extent of tumor immune infiltration and stromal cells has significant value for tumor diagnosis and prognosis evaluation. This study provides a comprehensive analysis of TPPP3 expression in cancer databases and its relationship to the prognosis of cancer patients. Then, we performed pathological verification with clinical specimens. In addition, we also examined the relationship between TPPP3 and tumor-infiltrating immune cells in HNSC through the Tumor Immunity Estimation Resource 2.0(TIMER 2.0). This report clarifies the important role of TPPP3 in HNSC and provides evidence for the relationship between TPPP3 and tumor-immune cell infiltration interactions.

## 2. Results

### 2.1. TPPP3 mRNA Expression Levels in Different Types of Human Cancer

**Figure 1:**
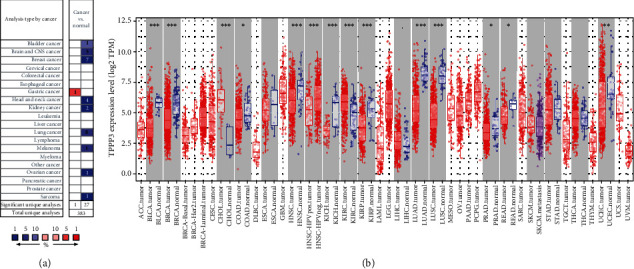
TPPP3 expression levels in different types of human tumors. (a) In the Oncomine database, TPPP3 expression in different cancer datasets compared to normal tissues. (b) Human TPPP3 expression levels in different tumor types from Timer. BRCA (breast invasive carcinoma), CESC (cervical squamous cell carcinoma and endocervical adenocarcinoma), CHOL (cholangiocarcinoma), COAD (colon adenocarcinoma), DLBC (Lymphoid Neoplasm Diffuse Large B-cell Lymphoma), ESCA (Esophageal carcinoma), GBM (Glioblastoma multiforme), HNSC (head and neck squamous cell carcinoma), KICH (kidney chromophobe), KIRC (kidney renal clear cell carcinoma), KIRP (kidney renal papillary cell carcinoma), LAML (Acute Myeloid Leukemia), LGG (Brain Lower Grade Glioma), LIHC (Liver hepatocellular carcinoma), LUAD (lung adenocarcinoma), LUSC (lung squamous cell carcinoma), MESO (Mesothelioma), OV (ovarian serous cystadenocarcinoma), PAAD (Pancreatic adenocarcinoma), PCPG (Pheochromocytoma and Paraganglioma), PRAD (Prostate adenocarcinoma), READ (Rectum adenocarcinoma), SARC (sarcoma), SKCM (Skin Cutaneous Melanoma), STAD (Stomach adenocarcinoma), TGCT (Testicular Germ Cell Tumors), THCA (Thyroid carcinoma), THYM (Thymoma), UCEC (Uterine Corpus Endometrial Carcinoma), UCS (Uterine Carcinosarcoma), and UVM (Uveal Melanoma).

To understand the differences in TPPP3 expression between human tumor and nontumor tissues, we utilized the Oncomine database to analyze the expression levels of TPPP3 in multiple cancer types and different tumors and normal tissues. Comparing to normal tissues, the study demonstrated that TPPP3 observed lower expression in bladder cancer, brain cancer, breast cancer, head and neck cancer, kidney cancer, lung cancer, melanoma, ovarian cancer, and sarcoma and high expression in gastric cancer (Figure 1(a)). To more accurately assess TPPP3 expression in human cancers, we used RNA-seq data from 31 malignancies in The Cancer Genome Atlas (TCGA) and Genotype-Tissue Expression (GTEx) to examine the TPPP3 expression.  Figure 1(b) shows the differential expression of TPPP3 between tumors and normal tissues. The TPPP3 expression was significantly decreased in HNSC, bladder urothelial carcinoma (BLCA), breast invasive carcinoma (BRCA), kidney chromophobe (KICH), kidney renal papillary cell carcinoma (KIPC), lung adenocarcinoma (LUAD), and lung squamous cell carcinoma (LUSC). However, compared with adjacent normal tissues, the TPPP3 expression was significantly higher in cholangiocarcinoma (CHOL) and kidney renal clear cell carcinoma (KIRC).

### 2.2. Prognostic Potential of TPPP3 in Cancers

**Figure 2:**
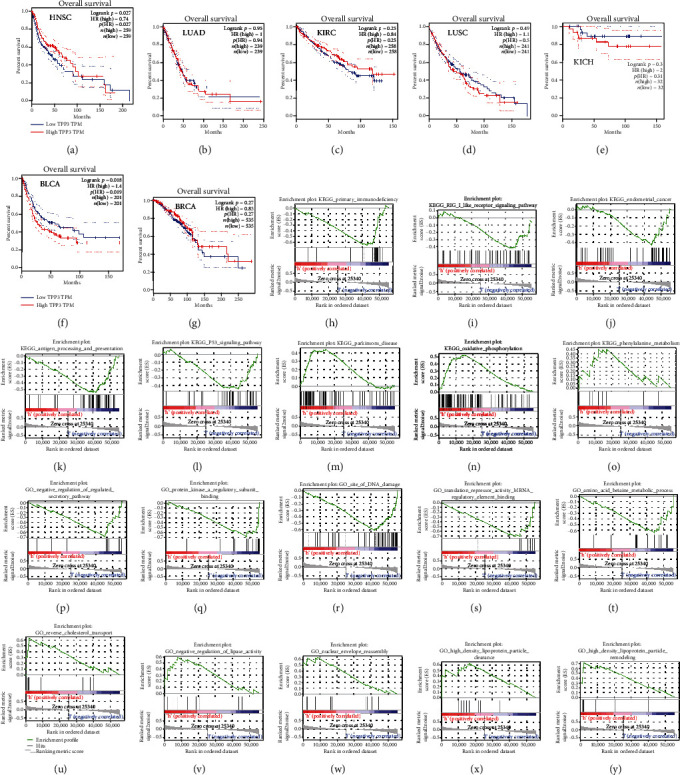
(a–g) Kaplan-Meier survival curves comparing the high and low expression of TPPP3 in different types of tumors by GEPIA. (h–y) list the most common functional gene sets enriched in HNSC samples with low and high expression of TPPP3.

To understand the prognosis of TPPP3 in tumors, Gene Expression Profiling Interactive Analysis (GEPIA) was used to determine whether TPPP3 expression is correlated with the prognosis of HNSC, LUAD, LUSC, KICH, BRCA, KIRC, and BLCA. (Figures 2(a)–2(g)) Notably, the low expression of TPPP3 affects the prognosis of HNSC (*p* = 0.027, HR = 0.74).

### 2.3. TPPP3 Gene Set Enrichment Analyses

To discover the underlying mechanism of TPPP3 and HNSC, Gene Set Enrichment Analysis (GSEA) was utilized to obtain the TPPP3-related gene collection based on the actual overall trend analysis and compare enrichment analysis such as Kyoto Encyclopedia of Genes and Genomes (KEGG) and Gene Ontology (GO). Consequently, the enrichment of 100 functional gene sets was obtained. The pathways closely related to the low expression of TPPP3 are “Antigen Processing and Presentation,” “Primary Immunodeficiency,” “RIG I Like Receptor Signaling Pathway,” “Endometrial Cancer,” and “P53 Signaling Pathway.” The pathways associated with TPPP3 high expression are “Parkingson Disease,” “Oxidative Phosphorylation,” and “Phenylalanine Metabolism” (Figures 2(h)–2(o)). “Translation repressor activity mRNA regulatory element-binding,” “Negative regulation of regulated secretory pathway,” “Negative regulation of regulated secretory pathway,” “Amino acid betaine metabolic process,” “Protein kinase a regulatory subunit binding,” and “Site of DNA damage” are the six most enriched items in GO related to the low expression of TPPP3 (Figures 2(p)–2(t)). The GO items associated with TPPP3 high expression are “Nuclear envelope reassembly,” “High density lipoprotein particle clearance,” “High density lipoprotein particle remodeling,” “Reverse cholesterol transport,” and “Negative regulation of lipase activity” (Figures 2(u)–2(y)).

### 2.4. TPPP3 Expression Is Associated with Immune Infiltration Levels in HNSC

**Figure 3:**
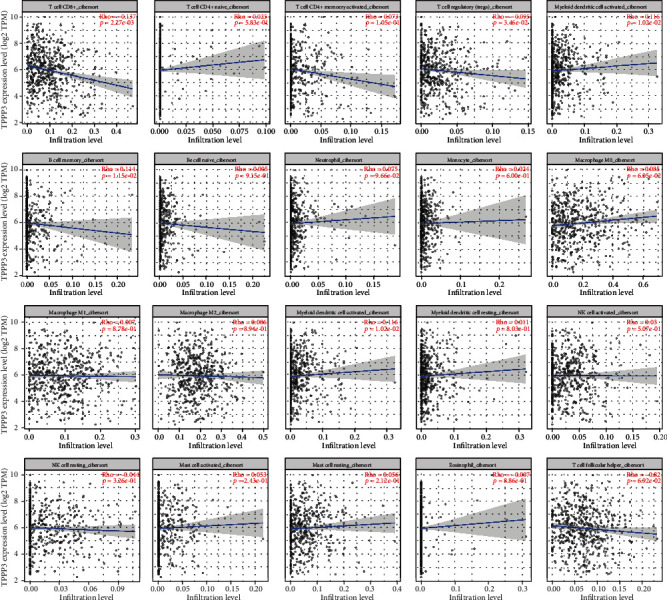
The correlation of TPPP3 expression with immune infiltration level in HNSC. TPPP3 expression is negatively correlated with infiltrating levels of CD8+ T cell, B cell, and myeloid dendritic cell. The infiltration level for each SCNA category is compared with the normal using a two-sided Wilcoxon rank-sum test.

Tumor-infiltrating lymphocytes are independent risk factors affecting tumor prognosis [9]. Therefore, we analyzed by TIMER2.0 whether the TPPP3 expression was associated with immune infiltration levels in HNSC. The results show that the TPPP3 expression has a certain correlation with the infiltration of CD8 + T cells, B cells, and myeloid dendritic cells (Figure 3).

### 2.5. Analysis of the Correlation between TPPP3 Expression and Immune Cell Genes

**Figure 4:**
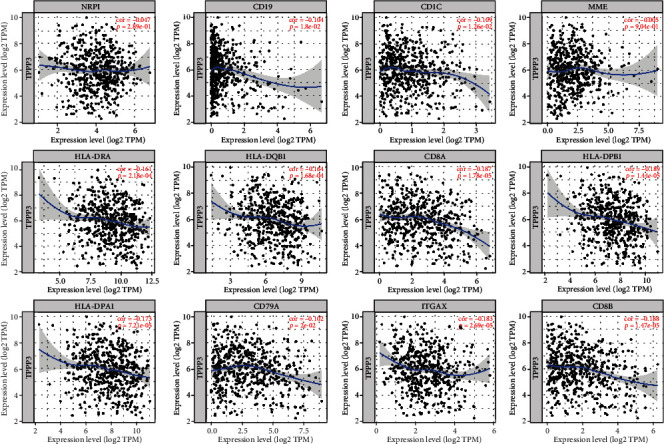
Scatterplots of correlations between TPPP3 expression and gene markers of CD8+T cells, B cell, and myeloid dendritic cell.

To gain insight into the intrinsic link between TPPP3 and related immune cells, we applied the TIMER 2.0 database to analyze the correlation between TPPP3 genes in HNSC related immune infiltrating cells. We obtained results on the correlation between TPPP3 expression and marker genes of tumor infiltration-associated immune cells, including CD8+T cells (CD8A and CD8B), B cell (CD19 and CD79a), and Myeloid dendritic cell (HLA-DPB1, HLA-DQB1, HLA-DRA, HLA-DPA1, CD1C, NRP1, and ITGAX). The results manifested that TPPP3 expression levels were negatively correlated with most of the immune marker genes in HNSC (Figure 4). In the high-level immune infiltration mode of HNSC, TPPP3 has an intense correlation with CD8 + T cell marker and DC markers. These results further indicate that there is a certain relationship between TPPP3 and DC penetration. Promote dendritic cells to regulate cytotoxic CD8 + T cell responses and exert anticancer effects [10, 11]. Whether TPPP3 is a key factor in mediating CD8 + T cell, DC, and tumor metastasis need further research.

### 2.6. Low Expression of TPPP3 in Clinical Specimens of NPC

**Figure 5:**
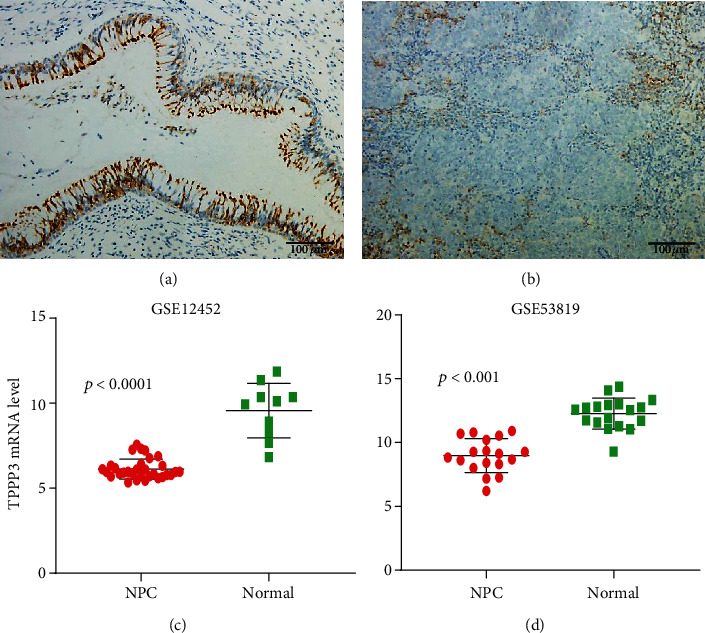
Expression of TPPP3 in nasopharyngeal carcinoma and nontumor epithelial tissue. (a) Nontumor epithelial tissue. (b) NPC tissue. (c) Analysis of TPPP3 expression levels in patients with NPC and normal tissues from GEO datasets GSE12452. *p* < 0.0001. (d) Analysis of TPPP3 expression levels in patients with NPC and normal tissues from GEO datasets GSE53819. *p* < 0.0001.

In the present research, the expression of TPPP3 in nasopharyngeal carcinoma (NPC) tissues was detected in 57 cases of NPC and 30 cases of normal tissue by immunohistochemistry, and the relationship between TPPP3 and clinicopathological features of NPC was analyzed at the same time. The results showed that the expression of TPPP3 in NPC was significantly lower than that in normal nasopharyngeal tissue; the difference was statistically significant (Figure 5, Table 1). But there was no significant correlation with clinicopathological features (Table 2).

## 3. Discussion

A growing number of researchers have been exploring the relationship between TPPP3 and tumor development in recent years. According to the Zhou et al.'s study, knockdown of TPPP3 inhibits cell proliferation of HeLa cells while inducing cell cycle arrest [8]. Li et al. demonstrated that TPPP3 was highly expressed in nonsmall cell lung cancer, and that the high expression of TPPP3 was positively correlated with clinical stage, tumor volume, lymph node metastasis, and poor prognosis [12]. Ye et al. also revealed that TPPP3 was highly expressed in colorectal cancer and was associated with colorectal cancer progression and poor prognosis, and that interfering with TPPP3 expression suppressed tumor cell proliferation, migration, and invasion, and increased apoptosis [13]. Although TPPP3 has not been extensively studied, according to the available data, it is found that the expression level of TPPP3 was increased in some tumors and correlated with tumor proliferation. The expression of TPPP3 is upregulated in many tumors, and experimental studies have confirmed its oncogenic effect. We carried out a pan-cancer analysis of TPPP3, which indicates that TPPP3 has a different expression trend with other tumors in HNSC. However, no relevant studies were found in head and neck squamous carcinoma. Therefore, it is necessary to carry out detailed research on TPPP3 in HNSC.

In this article, we first performed a pan-cancer analysis of TPPP3 expression. The Oncomine database and GEPIA were used to analyze the mRNA expression data of 31 tumors to check the expression level and prognosis of TPPP3 in different types of tumors. According to the results of the Oncomine database, we found that TPPP3 was lower expressed in breast cancer, head and neck cancer, kidney cancer, ovarian cancer, and sarcoma compared to normal tissues, while higher expression in gastric cancer. Furthermore, GEPIA demonstrated that compared with neighboring normal tissues, the expression of TPPP3 in HNSC, BLCA, BRCA, KICH, KIRC, LUAD, and LUSC was significantly reduced, while the expression of TPPP3 in CHOL and KIRC was significantly higher. We found that using different databases, TPPP3 expression levels differed among different tumor types, which may be due to the data collection method and the different biological characteristics of TPPP3. Interestingly, in these databases, we found consistency in TPPP3 expression in head and neck squamous cell carcinoma. In addition, the analysis of survival data indicates that low levels of TPPP3 expression are associated with a poor prognosis for HNSC.

In this research on the mechanism of TPPP3, we selected the transcript data of 70 cases HNSC in the TCGA database and performed GSEA analysis on these datasets. The pathways closely related to the low expression of TPPP3 are “Antigen Processing and Presentation,” “Primary Immunodeficiency,” “RIG I Like Receptor Signaling Pathway,” “Endometrial Cancer,” and “P53 Signaling Pathway.” Results also indicate that TPPP3 may be related to the mechanism of tumor immunity. Therefore, we shifted the research focus of TPPP3 to research on tumor immune infiltration. Our results indicate that TPPP3 expression is correlated with multiple immune cell infiltration levels in head and neck squamous cell carcinoma. There is a negative correlation between the expression of TPPP3 and the infiltration levels of CD8+T cells and B memory cells. However, TPPP3 expression is positively correlated with DC cells. Moreover, the correlation between TPPP3 expression and immune cell marker genes suggests that TPPP3 is regulating the immunological role of HNSC. Further, the genetic markers of CD8+ T cells and myeloid dendritic cells are weakly correlated with TPPP3 expression. These results reveal a potential regulatory role of TPPP3 in tumor-associated myeloid dendritic cell-mediated T cell toxic effects.

In addition, to further understanding of TPPP3 expression in head and neck tumors, we also performed immunohistochemistry on clinical NPC specimens and validated tumor and normal tissue TPPP3 mRNA expression in two GEO NPC datasets. The results we obtained are consistent with those of Oncomine and GEPIA in that TPPP3 expression is significantly reduced in the HNSC. But there is no significant correlation with clinicopathological features. This study for the first time analyzed the expression and prognosis of TPPP3 in head and neck squamous cell carcinoma, unexpectedly, in contrast to the expression in other tumors. We are as well the first to reveal the link between TPPP3 and immune infiltration. This study also has some shortcomings, such as we still need to perform in vitro and in vivo experiments on TPPP3 for cellular function studies as well as pathway studies.

## 4. Materials and Methods

### 4.1. Clinical Specimens

NPC tissues from 57 confirmed and untreated NPC patients (median age: 45 years old; female: *n* = 16; male: *n* = 41) were collected from the First Affiliated Hospital of Guangxi Medical University in 2016-2019. The diagnoses were according to the World Health Organization (WHO) classification. And 30 nontumor nasopharyngeal tissues obtained by fiber optic nasopharyngoscopy and tonsillectomy were used as controls. Informed consent was obtained from all donors involved, and ethical approval for this study was granted by the Ethical Review Committee of the First Affiliated Hospital of Guangxi Medical University.

### 4.2. Oncomine Database Analysis

Oncomine (https://www.oncomine.org/resource/login.html) [14] is the world's largest cancer gene microarray database and integrated data-mining platform, which can be used to analyze gene expression differences, search for outliers, and predict coexpression genes. The Oncomine database was utilized to determine the expression level of the TPPP3 gene in various types of cancer. Threshold determinations were set as follows: *p* value of 0.001, fold change of 1.5, and gene ranking of all.

### 4.3. GEPIA Website Analysis

GEPIA (http://gepia.cancer-pku.cn/index.html) [15] is a newly created interactive web server for analyzing RNA sequencing expression data of tumor and normal samples from the TCGA and GTEx projects. GEPIA was employed to analyze the expression of the TPPP3 gene in 33 different types of tumors and generate survival curves including overall survival (OS) and disease-free survival (DFS). The significantly related genes were further confirmed by GEPIA, and the correlation coefficient was determined by Spearman's method.

### 4.4. TCGA Database

TCGA is the world's largest cancer database, which includes clinical data, genomic variation, mRNA expression, miRNA expression, methylation, and other data on various human cancers. The mRNA expression data of 70 HNSC and 13 adjacent control samples and relevant clinical data were downloaded from the TCGA database (https://portal.gdc.cancer.gov) to further study. Clinical data information was evaluated according to the TNM staging requirements of the 8th edition of the Joint Committee on Cancer [16].

### 4.5. Gene Set Enrichment Analyses

Gene Set Enrichment Analyses (GSEA) [17] is a tool for transcriptomic data that determines the enrichment of gene sets based on the correlation between gene expression data and the phenotype. First, RNA-sequencing data of 70 HNSC samples from the TCGA were divided into two groups, high and low expression, according to the TPPP3 median value of gene expression. Then, the annotated gene sets (c2.cp.KEGG.v7.0.symbols.gmt) and (c5.all.v6.2.symbols.gmt) were selected as reference gene sets for GSEA. Cut-off criteria were defined as *p* < 0.05, FDR < 0.05, and enrichment score (ES) > 0.6.

### 4.6. TIMER Website Analysis

TIMER2.0 (http://timer.cistrome.org) [18] is an interactive web server for systematically estimating the abundance of immune infiltration in tumors from the TCGA database. We performed a genetic module to analyze the correlation between TPPP3 expression and immune infiltration abundance in HNSC, including CD4+ T cells, CD8+ T cells, B cells, neutrophils, Monocyte, Macrophages, Common lymphoid progenitor, NK cell, Endothelial cell, Mast cell, Tregs, and dendritic cells, via gene modules. These genetic markers have been cited in previous researches [19, 20]. The website provides both TIMER and CIBERSORT immune infiltration estimations.

### 4.7. Immunohistochemistry

NPC tissues were fixed in formalin and embedded in paraffin. First, 6 × 8 tissue blocks were created, and 4 *μ*m thick tissue sections were then cut and placed on glass slides. After blocking, slides were incubated at 4°C in a humidified chamber overnight with a monoclonal anti-TPPP3 antibody (GeneTex, USA) at a 1 : 200 dilution. A biotinylated secondary antibody and horseradish peroxidase-labeled avidin were subsequently applied. The diaminobenzidine method was used for visualization. The expression levels of proteins in the NPC and adjacent normal tissue sections were observed according to the staining pattern.

### 4.8. GEO Database Analysis

GEO (https://www.ncbi.nlm.nih.gov/geo/) is a gene expression database created and maintained by the National Center for Biotechnology Information (NCBI) in the United States. GSE12452 [21] and GSE53819 [22] datasets were obtained from the GEO database. The GSE12452 dataset contained 31 nasopharyngeal carcinomas and 10 normal healthy nasopharyngeal tissue specimens. GSE53819 contained data from 18 NPC samples and 18 noncancerous nasopharyngeal tissue samples. TPPP3mRNA expression levels were compared between the tumor group and normal tissues in these two datasets.

### 4.9. Statistical Analysis

The mRNA expression between NPC and normal group was compared, respectively, using *t*-test. The correlation of TPPP3 expression was evaluated by Spearman's correlation and statistical significance. *p* values < 0.05 were considered statistically significant.

## 5. Conclusions

All in all, the TPPP3 expression is significantly reduced in HNSC, and the low expression of TPPP3 is associated with CD8 + T cell and myeloid dendritic cell immune infiltration levels. Therefore, thus, TPPP3 can serve as a potential prognostic indicator for HNSC patients and may play an important role in immune cell infiltration.

## Figures and Tables

**Table 1:** Immunohistochemical staining showed that TPPP3 in nasopharyngeal carcinoma was lower than that in normal nasopharyngeal mucosa.

Group	Positive (+)	Negative (-)	Sum	Positive rate (%)	*p* value
Nasopharyngeal carcinoma	7	50	57	12.28	<0.001
Normal nasopharyngeal mucosa	27	3	30	90.00	

**Table 2:** The relationship between the expression of TPPP3 and the clinicopathological features of nasopharyngeal carcinoma.

Group	*n*	Negative (-)	Positive (+)	Positive proportion (%)	*p* value
Gender					
Male	41	36	5	12.20	>0.05
Female	16	14	2	12.50	
Age					
≤45	25	24	3	12.00	>0.05
>45	32	28	4	12.50	
Differentiation or not					
Differentiated	6	5	1	16.67	>0.05
Undifferentiated	51	45	6	11.76	
T stage					
T1, T2	21	19	2	9.52	>0.05
T3, T4	36	31	5	13.89	
N stage					
≥N1	52	46	6	11.54	>0.05
N0	5	4	1	20.00	
M stage					
M0	49	43	6	12.24	>0.05
M1	8	7	1	12.50	
Clinical stage					
I, II	5	4	1	20.00	>0.05
III, IV	52	46	6	11.54	

## Data Availability

The data used in this study are from open public databases, and how to obtain them has been explained in the manuscript.

## Abbreviations

TPPP3: Tubulin polymerization promoting protein family member 3
GSEA: Gene expression profiling interactive analysis
TIMER: Tumor immune estimation resource
GEPIA: Gene expression profiling interactive analysis
TCGA: The Cancer Genome Atlas
GTEx: The genotype-tissue expression
HNSC: Head and neck squamous carcinoma
MT: Microtubule
BLCA: Bladder urothelial carcinoma
BRCA: Breast invasive carcinoma
KICH: Kidney chromophobe
KIRC: Kidney renal papillary cell carcinoma
LUAD: Lung adenocarcinoma
LUSC: Lung squamous cell carcinoma
CHOL: Cholangiocarcinoma
NPC: Nasopharyngeal carcinoma
WHO: The World Health Organization
OS: Overall survival
DFS: Disease-free survival.
